# Cognitive function predicts all-cause mortality independent of social engagement among community-dwelling elderly Japanese men: a 15-year prospective cohort study

**DOI:** 10.1265/ehpm.26-00105

**Published:** 2026-08-27

**Authors:** Yuki Fujita, Masayuki Morikawa, Katsuyasu Kouda, Junko Tamaki, Nozomi Okamoto, Masayuki Iki

**Affiliations:** 1 Department of Hygiene and Public Health, Faculty of Medicine, Kansai Medical University, Hirakata, Osaka 573-1010, Japan; 2 Mie Prefectural Mental Care Center, Tsu, Mie 514-0818, Japan; 3 Department of Hygiene and Public Health, Faculty of Medicine, Osaka Medical and Pharmaceutical University, Takatsuki, Osaka 569-8686, Japan; 4 Graduate School of Education, Hyogo University of Teacher Education, Kato-City, Hyogo 673-1494, Japan

**Keywords:** Cognitive function, Elderly, Epidemiology, Man, Mortality

## Abstract

**Background:**

As the global population ages, cognitive impairment has become a growing public health concern. Although epidemiological studies have examined its association with mortality, sex-stratified analyses and community-based investigations in elderly Asian men remain scarce. Furthermore, social engagement may confound the association between cognitive impairment and mortality; yet few studies among community-dwelling elderly Asian men have simultaneously examined cognitive impairment and social engagement in relation to all-cause mortality in a prospective cohort design. This study therefore aimed to examine whether cognitive impairment is associated with an increased risk of all-cause mortality independent of social engagement among community-dwelling elderly Japanese men over a 15-year follow-up period.

**Methods:**

This prospective cohort study enrolled 2,174 men aged ≥65 years. Follow-up assessments were conducted at five, ten, and fifteen years, with all-cause mortality as the primary outcome. Cognitive function was assessed using the Mini-Mental State Examination. Hazard ratios (HRs) with 95% confidence intervals (CIs) were estimated using Cox proportional hazards models to examine the association between cognitive function and all-cause mortality. Confounder-adjusted Kaplan–Meier survival curves were generated using inverse probability of treatment weighting.

**Results:**

The final analytic sample comprised 1,741 men. High social engagement was associated with a lower risk of all-cause mortality. Compared with participants with normal cognitive function, the hazard ratios (HRs) for all-cause mortality were 1.29 (95% CI: 1.06–1.56) for mild cognitive impairment and 1.59 (95% CI: 1.10–2.29) for severe cognitive impairment, after adjusting for potential confounding factors including social engagement.

**Conclusions:**

Cognitive impairment independently predicts an increased risk of all-cause mortality in community-dwelling elderly Japanese men.

## Introduction

Japan is witnessing rapid population aging, with those aged 65 years and over accounting for 29.1% of the total population [1]. As the total population declines while the number of individuals aged 65 and over increases, the aging rate is projected to reach 33.3% in 2037 [2]. In this context, cognitive impairment is becoming an increasingly serious public health concern among older adults.

Previous studies have reported an association between cognitive impairment and increased all-cause mortality in older adults [3–6]. Notably, the association between cognitive function and all-cause mortality appears to vary by sex. A retrospective cohort study of patients with a diagnosis of dementia reported that men face a higher risk of all-cause mortality than women [7]. However, many epidemiological studies among community-dwelling older adults have not reported results stratified by sex, leaving this relationship insufficiently characterized. Sex-specific differences in mortality risk, lifestyle patterns, comorbidity profiles, and health-seeking behavior may substantially modify the relationship between cognitive function and survival outcomes [7]. Given this evidence of sex-specific vulnerability, a study focused exclusively on men offers the opportunity to elucidate the cognitive function–mortality relationship within this potentially high-risk subgroup.

Despite this growing body of evidence, several critical gaps in the literature remain unaddressed. First, although community-based prospective studies have examined the cognitive impairment–mortality association, existing investigations in Asian populations have notable methodological limitations. Some relied on health checkup–based recruitment rather than community sampling [3], others were conducted in non-Japanese populations [4, 6], and studies specific to Japanese men have been limited by small male-specific sample sizes or single-age cohort designs [5]. Consequently, community-based prospective investigations with adequate statistical power that focus specifically on elderly Japanese men remain lacking. Given that community-dwelling older adults have distinct health profiles, lifestyle patterns, and social circumstances compared with those in clinical or health checkup settings, the generalizability of existing findings to this population warrants further examination. Second, and central to the present study, although social engagement has been associated with both cognitive impairment and mortality [8–11], its potential role as a confounder of the association between cognitive function and all-cause mortality has rarely been accounted for in prior research. While a small number of studies have considered related associations [12, 13], the available evidence remains limited in both quantity and scope; to our knowledge, no study has explicitly examined social participation, cognitive impairment, and all-cause mortality together within a community-dwelling cohort of elderly men. Failure to adjust for social engagement may therefore lead to overestimation of the independent effect of cognitive impairment on mortality risk. These methodological advances are not merely statistical refinements: by isolating the independent contribution of cognitive impairment to mortality risk, this study provides more reliable evidence to inform risk stratification and preventive strategies targeted at community-dwelling elderly men. Accordingly, the present study aimed to examine whether cognitive impairment is independently associated with an increased risk of all-cause mortality, after adjustment for social engagement, among community-dwelling elderly Japanese men over a follow-up period of approximately 15 years.

## Methods

### Study participants

The Fujiwara-kyo Osteoporosis Risk in Men (FORMEN) study was conducted as a longitudinal study in community-dwelling elderly Japanese men. We recruited men aged ≥65 years from four cities in Nara Prefecture, Japan. The FORMEN study was originally designed to investigate osteoporosis-related outcomes in men, which is why it enrolled male participants only. This all-male cohort nonetheless offers an opportunity to examine the cognitive function–mortality association within a sex-specific framework, given the accumulating evidence that men may exhibit distinct vulnerability to cognitive impairment-related mortality [5]. A total of 2,174 individuals voluntarily enrolled in the study, all could walk independently, provided self-reported information, comprehended the study’s objectives, and gave written informed consent. Participants completed clinical examinations and a self-administered questionnaire at the baseline survey of the FORMEN study, which was conducted from October 2007 to October 2008 [14, 15]. Follow-up surveys were conducted with approximately five (February 2012 to November 2012), ten (February 2017 to March 2019), and fifteen (November 2022 to November 2023) years after the baseline survey. Of the initial 2,174 participants, 270 were excluded due to missing values for predictor variables or potential confounding factors, and 163 were excluded due to missing data on the date of death. Consequently, the final analysis included 1,741 men aged 65 to 94 years.

### Body size measurements

Height (cm) and body weight (kg) were measured using an automatic scale, with participants wearing no shoes and in light clothing (Tanita TBF-215; Tanita Inc., Japan). Body mass index (BMI, kg/m2) was computed as the quotient of weight and the square of height.

### Medical history and lifestyle factors

Participants completed a questionnaire covering medical histories of hypertension, diabetes mellitus, cardiovascular disease, and cerebrovascular disease, with responses recorded as either “yes” or “no”. The questionnaire also inquired about the use of medications for these conditions. Participants were instructed to bring their current medication records to the baseline visit, during which interviewers documented the names and dosages of the medications. In addition, participants completed a questionnaire addressing current smoking and drinking habits, habitual physical activity, spousal status (present/absent) and educational attainment. Education level was classified into two categories: “9 years or less” and “more than 9 years”, reflecting Japan’s compulsory 9-year education system, which encompasses elementary and junior high school. All interviews were conducted by trained public health nurses or physicians.

### Measurements of cognitive performance

The present study evaluated global cognitive function using the Mini-Mental State Examination (MMSE), which is widely used in epidemiological studies [16]. The MMSE comprises eleven items covering the following domains: time orientation (5 points), place orientation (5 points), registration of three words (3 points), mental calculation by serially subtracting seven from 100 (5 points), delayed recall of the three words presented earlier (3 points), naming objects (2 points), repeating a sentence (1 point), following a three-stage verbal instruction (3 points), reading and following a written instruction on a card (1 point), writing a sentence (1 point), and copying a figure on a sheet (1 point). These items can be grouped into six main cognitive subscales: orientation to time and place (10 points), registration (3 points), attention and calculation (5 points), recall (3 points), language (8 points) and constructional ability (1 point) [17]. The total MMSE score ranges from 0 to 30, with higher scores indicating better cognitive function. The severity of cognitive impairment was categorized into three groups: normal (28–30), mild cognitive impairment (MCI) (24–27) and severe cognitive impairment (SCI) (0–23) [18, 19].

### Assessment of higher-level functional capacity including social engagement

To assess higher-level functional capacity, we employed the Tokyo Metropolitan Institute of Gerontology Index of Competence (TMIG-IC) [20, 21], a widely validated 13-item multidimensional tool for community-dwelling older adults (total score range: 0–13). This instrument encompasses three domains: instrumental activities of daily living (five items), intellectual activity (four items), and social engagement (four items). Participants responded to each item dichotomously (“yes” = 1 point; “no” = 0 points), with higher scores indicating superior functional capability. The social engagement subscale was selected as the primary covariate of interest for three reasons. First, social engagement represents the dimension of higher-level functional capacity most closely aligned with social participation and interpersonal connectedness, both of which have been independently associated with mortality risk in older adults [8, 9]. Second, the social engagement subscale has been shown to constitute a statistically independent factor in confirmatory factor analyses of the TMIG-IC [22, 23]. Third, the instrumental activities of daily living and intellectual activity subscales of the TMIG-IC overlap substantially with the cognitive domains assessed by the MMSE, and their simultaneous inclusion in regression models would risk introducing multicollinearity and potentially destabilizing the coefficient estimates for cognitive function.

### Identification of deaths

Deaths occurring from baseline through the end of November 2023 were ascertained through follow-up mail and telephone contact with family members or other proxies of non-responding participants, as well as through inquiries to municipal resident registries for those who could not be reached.

### Statistical analysis

**Fig. 1:**
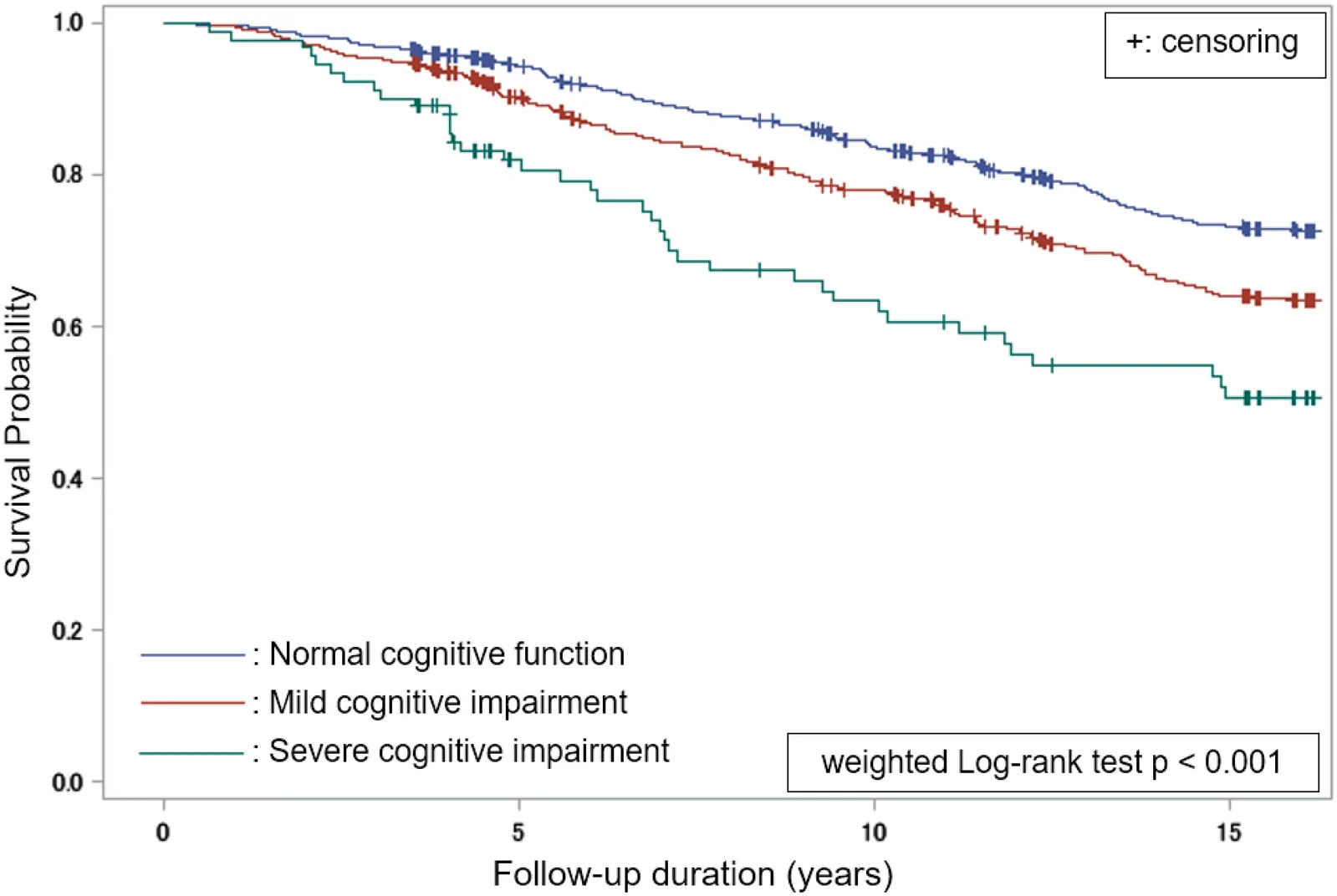
Kaplan–Meier unadjusted survival estimates stratified by baseline cognitive impairment severity.

**Fig. 2:**
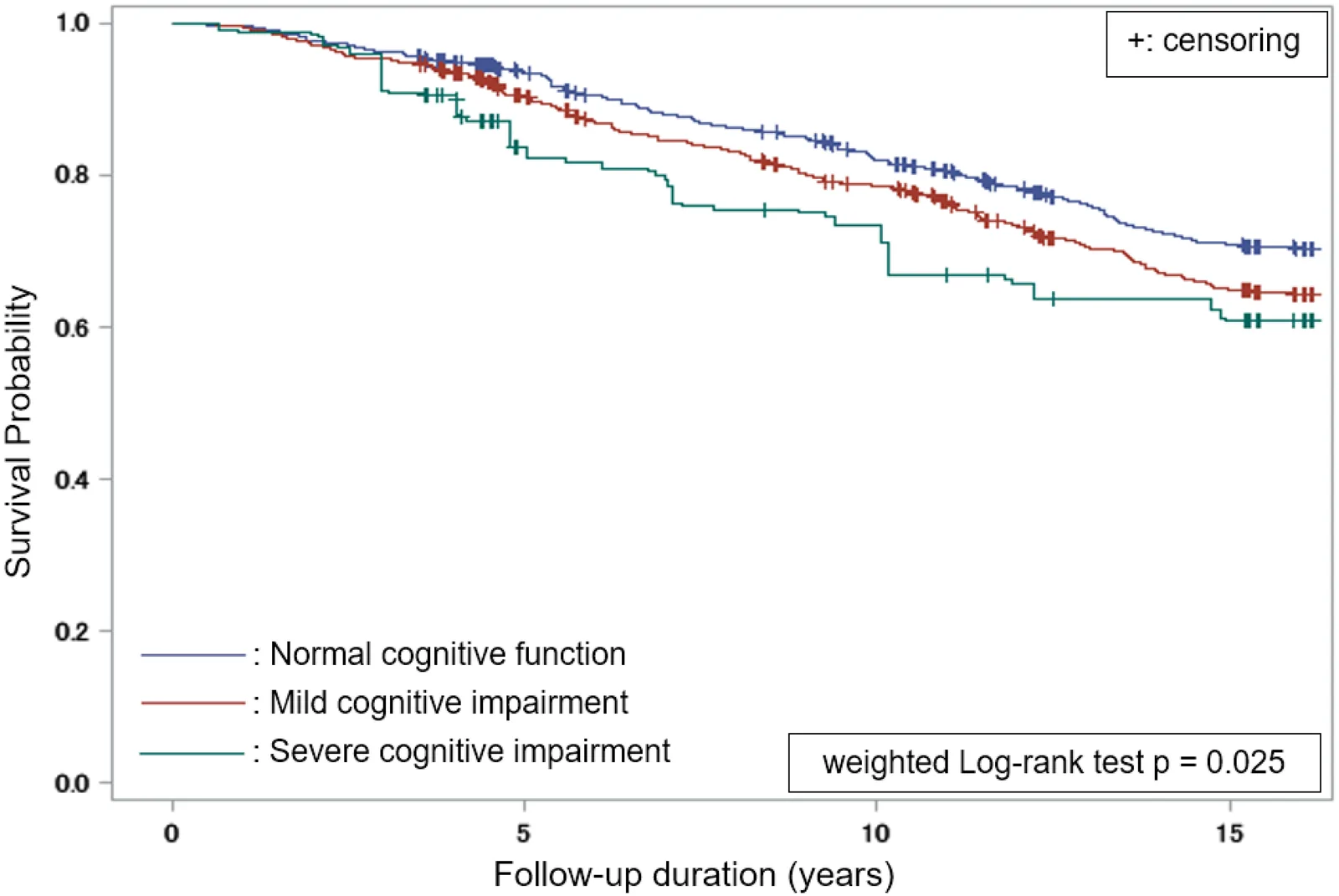
Kaplan–Meier adjusted survival estimates stratified by baseline cognitive impairment severity

Participants were classified into three categories based on MMSE score: normal (28–30), MCI (24–27), and SCI (0–23). One-way analysis of variance was used to compare means across categories, and Fisher’s exact test was used to compare proportions of categorical variables across categories (Table 1). Differences between survivors and deceased were assessed using the unpaired t-test for continuous variables (Table 2). Cox proportional hazards models were used to estimate hazard ratios (HRs) for all-cause mortality using three sets of exposure variables. First, HRs were estimated for each MMSE subscale, adjusting for the same set of confounding factors (Table 3). Second, unadjusted (crude) HRs were estimated separately for MMSE category and for social engagement score (Table 4). Third, HRs for MMSE category and social engagement were estimated using two sequentially adjusted models: Model 1 adjusted for cognitive function, social engagement, spousal status, age, and education, and Model 2 additionally adjusted for BMI, physical activity, habitual drinking, habitual smoking, and history of hypertension, dyslipidemia, type 2 diabetes mellitus, cardiovascular disease, cerebrovascular disease, and osteoporotic fracture (Table 5). In these models, follow-up time was calculated from the date of the baseline survey to the date of death. For participants who did not experience the outcome, follow-up time was censored at the date of dropout or the end of the follow-up period, whichever came first. Kaplan-Meier survival curves were constructed with inverse probability of treatment weighting (IPTW) adjustment to estimate survival probabilities for each MMSE category, and weighted log-rank tests were used to compare survival distributions between categories (Fig. 1 and Fig. 2). The IPTW method is a weighted analysis in which the weights are the inverse probability of assignment to the observed MMSE category, estimated from the propensity scores using multinomial logistic regression [24]. The propensity score model included the following baseline covariates: age, education, social engagement, spousal status, BMI, physical activity, habitual drinking, habitual smoking, history of hypertension, history of dyslipidemia, history of type 2 diabetes mellitus, history of cardiovascular disease, history of cerebrovascular disease and history of osteoporotic fracture. Predicted probabilities of membership in each of the three categories were obtained for every participant. Stabilized IPTW were then calculated as the ratio of the marginal probability of belonging to the observed group to the individual’s predicted probability of belonging to that group. Covariate balance across the three cognitive status groups was assessed before and after IPTW weighting using standardized mean differences (SMDs), calculated separately for each pairwise group comparison. For continuous covariates, SMDs were calculated using weighted means and variances; for categorical covariates (with multi-level variables represented as indicator variables), weighted proportions were used. An absolute SMD < 0.10 was considered to indicate adequate balance. A p-value of less than 0.05 was considered statistically significant. All statistical analyses were performed using SAS software (Version 9.4; SAS Institute, Cary, NC, USA).

**Table 1:** Characteristics of participants classified by MMSE category

	**Total** **(N = 1741)**			**MMSE category (MMSE total score)**									**p-value**

				**Normal (28–30)** **(N = 937)**			**MCI (24–27)** **(N = 713)**			**SCI (0–23)** **(N = 91)**			
At baseline	Mean		SD	Mean		SD	Mean		SD	Mean		SD	
MMSE total score	27.4	±	2.3	29.2	±	0.8	25.7	±	1.0	22.0	±	1.5	<0.01
Age (years)	73.0	±	5.2	72.5	±	5.0	73.3	±	5.3	74.8	±	5.4	<0.01
Height (cm)	162.9	±	5.7	163.4	±	5.7	162.5	±	5.8	160.9	±	5.9	<0.01
Weight (kg)	61.0	±	8.5	61.5	±	8.5	60.6	±	8.4	59.2	±	9.0	<0.05
BMI (kg/m^2^)	23.0	±	2.7	23.0	±	2.7	22.9	±	2.7	22.8	±	3.0	0.74
Physical activity (MetsMin/day)	260.8	±	256.3	249.5	±	236.5	276.2	±	279.4	256.3	±	260.0	0.11
Education (≤9 years), n (%)	433 (24.9)			151 (16.1)			227 (31.8)			55 (60.4)			<0.01
Drinking, n (%)	1091 (62.7)			608 (64.9)			432 (60.6)			51 (56.0)			0.08
Smoking, n (%)	285 (16.4)			146 (15.6)			116 (16.3)			23 (25.3)			0.07
Type 2 diabetes mellitus, n (%)	169 (9.7)			78 (8.3)			83 (11.6)			8 (8.8)			0.08
Cardiovascular disease, n (%)	106 (6.1)			61 (6.5)			42 (5.9)			3 (3.3)			0.53
Cerebrovascular disease, n (%)	109 (6.3)			47 (5.0)			51 (7.2)			11 (12.1)			<0.05
Hypertension, n (%)	734 (42.2)			391 (41.7)			300 (42.1)			43 (47.3)			0.59
Dyslipidemia, n (%)	299 (17.2)			157 (16.8)			131 (18.4)			11 (12.1)			0.30
Osteoporotic fracture, n (%)	60 (3.4)			31 (3.3)			26 (3.6)			3 (3.3)			0.95
Social engagement, n (%)													
0 point (low)	11 (0.6)			4 (0.4)			3 (0.4)			4 (4.4)			<0.01
1 point	51 (2.9)			29 (3.1)			21 (3.0)			1 (1.1)			
2 points	152 (8.7)			84 (9.0)			59 (8.3)			9 (9.9)			
3 points	303 (17.4)			162 (17.2)			124 (17.4)			17 (18.7)			
4 points (high)	1224 (70.3)			658 (70.2)			506 (71.0)			60 (65.9)			
Spousal status: present, n (%)	1602 (92.0)			874 (93.3)			648 (90.9)			80 (87.9)			0.06
During the follow-up													
Deceased, n (%)	486 (27.9)			225 (24.0)			222 (31.1)			39 (42.9)			<0.01
Follow-up times (years)	11.9	±	4.9	12.5	±	4.6	11.4	±	5.0	10.0	±	5.4	<0.01

BMI, body mass index; MMSE, Mini-Mental State Examination; MCI, mild cognitive impairment; SCI, severe cognitive impairment

Continuous variables were compared using one-way analysis of variance; categorical variables were compared using Fisher’s exact test.

**Table 2:** Mean score of the MMSE total and subscales

	**Total** **(N = 1741)**	**Survivors** **(N = 1255)**	**Deceased** **(N = 486)**	**p-value**
MMSE total score	27.39 ± 2.28	27.57 ± 2.18	26.93 ± 2.46	<0.01
MMSE subscale score				
Orientation to time and place	9.67 ± 0.66	9.71 ± 0.59	9.59 ± 0.79	<0.01
Registration	2.99 ± 0.11	2.99 ± 0.11	2.99 ± 0.10	0.69
Attention and calculation	3.49 ± 1.70	3.56 ± 1.68	3.29 ± 1.75	<0.01
Recall	2.53 ± 0.68	2.58 ± 0.63	2.38 ± 0.79	<0.01
Language	7.74 ± 0.56	7.75 ± 0.55	7.72 ± 0.61	0.29
Constructional ability	0.97 ± 0.17	0.97 ± 0.16	0.96 ± 0.19	0.19

MMSE, Mini-Mental State Examination

P-values were calculated using the unpaired t-test.

**Table 3:** Adjusted HRs of all-cause mortality for each MMSE subscale

	**MMSE subscale**	**HR**	**95% CI**
Model 1	Orientation to time and place	1.20	1.06–1.36
	Registration	0.72	0.35–1.49
	Attention and calculation	1.06	1.00–1.11
	Recall	1.18	1.05–1.33
	Language	1.13	0.97–1.31
	Constructional ability	1.15	0.72–1.82
Model 2			
	Orientation to time and place	1.17	1.04–1.33
	Registration	0.64	0.30–1.36
	Attention and calculation	1.05	0.99–1.11
	Recall	1.17	1.04–1.31
	Language	1.11	0.96–1.29
	Constructional ability	1.29	0.81–2.04

HR, hazard ratio; MMSE, Mini-Mental State Examination; CI, confidence interval

HRs were estimated using Cox proportional hazards models.

HR: hazard ratio per 1-unit decrease.

Model 1: Adjusted for age, education.

Model 2: Adjusted for age, education, social engagement, spousal status, BMI, physical activity, habitual drinking, habitual smoking, history of hypertension, history of dyslipidemia, history of type 2 diabetes mellitus, history of cardiovascular disease, history of cerebrovascular disease and history of osteoporotic fracture

**Table 4:** Unadjusted HRs of all-cause mortality according to MMSE category and social engagement

	**Person-years** **(PY)**	**Death** **(N)**	**Mortality rate** **(per 100 PY)**	**HR**	**95% CI**
MMSE category					
Normal (28–30)	11672.0	225	1.93	1.00	
MCI (24–27)	8163.2	222	2.72	1.43	1.19–1.73
SCI (0–23)	908.4	39	4.29	2.32	1.65–3.26
Social engagement					
0 point (low)	114.9	7	6.09	1.00	
1 point	590.3	21	3.56	0.57	0.24–1.34
2 points	1755.7	47	2.68	0.43	0.19–0.95
3 points	3613.2	78	2.16	0.34	0.16–0.75
4 points (high)	14669.5	333	2.27	0.36	0.17–0.76

HR, hazard ratio; MCI, mild cognitive impairment; SCI, severe cognitive impairment; CI, confidence interval; N, number

HRs were estimated using Cox proportional hazards models.

**Table 5:** Adjusted HRs of all-cause mortality according to MMSE category and social engagement

		**Mortality rate** **(per 100 PY)**	**HR**	**95% CI**
Model 1	MMSE category			
	Normal (28–30)	1.93	1.00	
	MCI (24–27)	2.72	1.27	1.05–1.53
	SCI (0–23)	4.29	1.77	1.24–2.54
	Social engagement			
	0 point (low)	6.09	1.00	
	1 point	3.56	0.68	0.29–1.64
	2 points	2.68	0.52	0.23–1.16
	3 points	2.16	0.37	0.17–0.82
	4 points (high)	2.27	0.47	0.22–1.00
Model 2	MMSE category			
	Normal (28–30)	1.93	1.00	
	MCI (24–27)	2.72	1.29	1.06–1.56
	SCI (0–23)	4.29	1.59	1.10–2.29
	Social engagement			
	0 point (low)	6.09	1.00	
	1 point	3.56	0.59	0.25–1.43
	2 points	2.68	0.50	0.22–1.14
	3 points	2.16	0.34	0.15–0.74
	4 points (high)	2.27	0.43	0.20–0.92

HR, hazard ratio; MMSE, Mini-Mental State Examination; MCI, mild cognitive impairment; SCI, severe cognitive impairment; CI, confidence interval; PY, Person-years; N, number

HRs were estimated using Cox proportional hazards models.

Model 1 includes cognitive function, social engagement, spousal status, age, and education as explanatory variables.

Model 2 includes cognitive function, social engagement, spousal status, age, education, BMI, physical activity, habitual drinking, habitual smoking, history of hypertension, history of dyslipidemia, history of type 2 diabetes mellitus, history of cardiovascular disease, history of cerebrovascular disease, and history of osteoporotic fracture as explanatory variables.

## Results

The mean age of the 1,741 participants at baseline was 73.0 years. At baseline, 41.0% (n = 713) and 5.2% (n = 91) of participants were classified as MCI and SCI, respectively. A total of 486 deaths were identified over a mean follow-up period of 11.9 years (20,743.6 person-years [PY]), corresponding to a mortality rate of 2.34 per 100 PY.

Table 1 shows the baseline characteristics of participants according to the three MMSE categories defined by MMSE total score. Participants with lower MMSE scores were older and had lower educational attainment. Regarding social engagement, the proportion of participants in the lowest category (0 points) was notably higher in the SCI group (4.4%) than in the Normal (0.4%) and MCI (0.4%) groups, while the proportion in the highest category (4 points) was somewhat lower in the SCI group (65.9%) compared with the Normal (70.2%) and MCI (71.0%) groups.

Table 2 shows the mean MMSE scores stratified by survival status. Deceased participants had significantly lower scores in total score, orientation to time and place, attention and calculation, and recall compared with survivors.

Table 3 shows the hazard ratios (HRs) of all-cause mortality for each subscale of the MMSE. The full adjusted HRs for mortality were 1.17 (95% CI: 1.04–1.33) for orientation to time and place, 1.05 (95% CI: 0.99–1.11) for attention and calculation, and 1.17 (95% CI: 1.04–1.31) for recall.

Table 4 shows the HRs for all-cause mortality according to MMSE total score or social engagement. The HRs for all-cause mortality were 1.43 (95% CI: 1.19–1.73) for MCI and 2.32 (95% CI: 1.65–3.26) for SCI. In addition, the HRs for all-cause mortality were 0.36 (95% CI: 0.17–0.76) for highest social engagement. Compared with participants with normal cognitive function, the HRs for all-cause mortality were 1.29 (95% CI: 1.06–1.56) for MCI and 1.59 (95% CI: 1.10–2.29) for SCI, after adjustment for potential confounding factors including social engagement (Table 5).

Before IPTW weighting, several covariates showed notable imbalance across the three MMSE categories, most markedly for education level (absolute SMD up to 1.02 for the Normal vs. SCI comparison). After weighting, covariate balance improved substantially overall: balance between the Normal and MCI groups was excellent, with all SMDs below 0.02, and among comparisons involving the SCI group, most covariates achieved adequate balance (absolute SMD < 0.10) after weighting, including education level, which improved from an absolute SMD of approximately 1.0 to below 0.10. However, a small number of covariates, including physical activity, history of type 2 diabetes mellitus, history of osteoporotic fracture, and one category of social engagement, retained residual imbalance after weighting (absolute SMD approximately 0.10–0.23).

Figure 1 shows Kaplan–Meier survival curves stratified by baseline cognitive impairment severity. In the unadjusted analysis, cumulative survival was lowest in the SCI group, followed by the MCI group and the normal cognition group, with a statistically significant difference among the three groups (weighted log-rank test, p < 0.001). Adjusted Kaplan-Meier factors curves, accounting for age, education, social engagement, spousal status, BMI, physical activity, habitual drinking, habitual smoking, and histories of hypertension, dyslipidemia, type 2 diabetes mellitus, cardiovascular disease, cerebrovascular disease, and osteoporotic fracture, showed statistically significant differences among the three groups (weighted log-rank test, p = 0.025) (shown in Fig. 2).

## Discussion

In this community-based prospective cohort study, we investigated the association between cognitive function assessed by the MMSE and all-cause mortality independent of social engagement in elderly Japanese men. Compared with men with normal cognitive function, those with cognitive impairment had a significantly increased risk of all-cause mortality after adjustment for potential confounders, including age, education, social engagement, BMI, physical activity, habitual drinking, habitual smoking, and histories of hypertension, dyslipidemia, type 2 diabetes mellitus, cardiovascular disease, cerebrovascular disease, and osteoporotic fracture. Among the three groups, men with SCI had the lowest survival rates, followed by those with MCI.

Regarding research on men examining the impact of cognitive impairment on mortality, a meta-analysis published in 2022 focused on the sex-specific associations of cognitive impairment assessed by the MMSE with all-cause mortality risk in older adults from the general population [25].

Although this meta-analysis does not include studies on Japanese men, it includes studies on men from other Asian countries (South Korea and China), both of which reported significantly elevated mortality risk among men with cognitive impairment compared with normal cognitive function [26, 27]. This meta-analysis further reported a pooled multivariable-adjusted relative risk of 1.34 (95% CI: 1.24–1.44) for all-cause mortality associated with cognitive impairment in men [25]. Our findings are therefore consistent with those of previous studies on the association between cognitive impairment assessed by the MMSE and all-cause mortality in community-dwelling elderly men.

Several factors may mediate the relationship between cognitive decline and mortality. Previous studies have demonstrated that older adults with cognitive impairment exhibit significantly lower Short Physical Performance Battery (SPPB) scores—comprising assessments of standing balance, lower extremity strength, and gait speed—compared with cognitively intact individuals, suggesting that cognitive decline may substantially impair overall physical functioning [28]. Furthermore, cognitive decline appears to be a major determinant of health literacy deterioration [29]. Diminished health literacy, in turn, may adversely affect medication adherence, reduce utilization of preventive healthcare services, and impair disease self-management capacity, potentially contributing to poor health outcomes and increased mortality risk. Beyond these general mechanisms, several male-specific pathways may further explain the observed association in this cohort of elderly men. First, men exhibit a distinct cardiovascular risk profile compared with women, characterized by earlier onset of atherosclerosis, higher prevalence of subclinical vascular disease, and greater susceptibility to cerebrovascular pathology [30, 31]. Accordingly, vascular cognitive impairment driven by cerebral small vessel disease and white matter lesions may progress more rapidly in men than in women. In addition, the decline in testosterone levels with aging in men has been associated with increased risk of both cognitive impairment and cardiovascular mortality, potentially representing a shared biological substrate linking cognitive decline to survival outcomes specifically in men [32, 33]. In addition, cognitive impairment also frequently co-occurs with physical frailty and unintentional weight loss, suggesting it may mark a broader frailty phenotype rather than act as a fully independent risk factor [34]. These mechanisms likely act synergistically with the pathways described above to elevate mortality risk in this cohort.

In the MMSE subscale analyses, orientation to time and place, attention and calculation, and recall were each independently associated with all-cause mortality after adjustment for the same potential confounders. Regarding MMSE subscales, previous studies using health checkup data and a population-based cohort in Korea have similarly reported that orientation, attention/calculation, and recall subscales are significantly associated with mortality risk [3, 4], consistent with our findings. Notably, declines in these specific subscales—orientation to time and place, attention and calculation, and recall—have also been implicated in health literacy deterioration [29], suggesting a potential mechanistic link between subscale-specific cognitive decline and mortality risk.

In the present study, social engagement was treated as a confounding variable and included as a covariate in the Cox proportional hazards models. This approach was based on the premise that social engagement may independently influence mortality risk and may also be associated with baseline cognitive function, thereby satisfying the conditions for confounding. However, it is also theoretically plausible that social engagement functions as a mediator on the causal pathway from cognitive decline to mortality—that is, cognitive impairment may lead to reduced social engagement, which in turn increases mortality risk. If this mediating role is operative, adjusting for social engagement in the Cox model would constitute over-adjustment, potentially leading to an underestimation of the total effect of cognitive impairment on mortality. Notably, the hazard ratios observed in the present study represent the direct effect of cognitive impairment on mortality after accounting for social engagement, and may therefore be conservative estimates of the total effect. Future studies employing causal mediation analysis frameworks would be valuable to formally decompose the total effect of cognitive impairment on mortality into direct and indirect (socially mediated) components.

These findings have several implications for clinical practice and public health policy. Given Japan’s rapidly aging population, incorporating cognitive screening into routine community health checkups for elderly men could facilitate earlier identification of individuals at elevated mortality risk and enable timely referral to preventive or supportive services. Furthermore, because the association between cognitive impairment and mortality persisted independent of social engagement, interventions focused solely on increasing social participation may be insufficient to mitigate mortality risk in this population; combined strategies addressing both cognitive health and other modifiable factors, such as physical activity and chronic disease management, may be warranted.

Several strengths of this study are worth noting. First, this was a prospective cohort study with a relatively large sample size and a long follow-up period, which allowed us to clearly establish the temporal sequence between exposure and outcome and provided sufficient statistical power to detect significant associations. Second, the single-center study design offers an advantage over multi-center designs in terms of quality control, as it eliminates variability arising from differences between institutions. Third, the study incorporates social engagement as a covariate, directly addressing the concern that prior studies have not disentangled the independent contribution of cognitive impairment to mortality from the role of social engagement. Fourth, it applies inverse probability of treatment weighting (IPTW) to Kaplan–Meier survival curves, enabling confounder-adjusted visualization of survival differences by cognitive impairment status, a methodological advance over unadjusted survival analyses in prior studies.

Several limitations of the present study should be acknowledged. First, the study sample was restricted to community-dwelling volunteers capable of independent ambulation, which may not be representative of the broader population within the corresponding age group. As such individuals are likely to be in comparatively better health than the general population, a healthy volunteer bias may have been introduced, potentially leading to an underestimation of the true magnitude of the associations examined. These factors may therefore limit the external validity and generalizability of the findings. Second, the inclusion of men only represents an important limitation of this study. Because the FORMEN study was originally designed to investigate osteoporosis-related outcomes in men, female participants were not recruited. As a result, the present findings cannot be generalized to women, and sex-stratified comparisons were not possible. Given that cognitive trajectories, social engagement patterns, and mortality risks differ between men and women, future studies that include both sexes with sex-stratified analyses would provide more comprehensive evidence. Third, as participants were recruited from a geographically limited region of Japan, the generalizability of the findings may be constrained. In particular, the results may not be directly applicable to populations of different ethnic backgrounds or those residing in other geographical settings, and cross-cultural applicability should be interpreted with caution. Fourth, although the MMSE is a widely used and well-validated instrument for assessing cognitive function, it has recognized limitations in sensitivity for detecting MCI in older adults [35]. Consequently, some individuals with MCI may have been misclassified as cognitively normal, potentially resulting in an underestimation of the true association between MCI and mortality. Nevertheless, the MMSE can be used to confirm the presence of cognitive impairment when it is clinically suspected [35]. In addition, dementia subtypes such as Alzheimer’s disease and vascular dementia could not be distinguished. Fifth, the absence of cause-specific mortality data represents a notable limitation, as it precludes a comprehensive understanding of the underlying conditions that may have contributed to the observed associations. Future studies incorporating cause-of-death information would allow more detailed investigation of the potential pathways linking cognitive impairment to mortality. Sixth, although IPTW weighting substantially improved covariate balance across the MMSE categories, residual imbalance remained for a small number of covariates in comparisons involving the SCI group, which may reflect limited overlap in propensity scores between this group and the others; this residual imbalance should be considered when interpreting the survival estimates involving the SCI group. Finally, given the observational nature of the study, the findings do not permit causal inference, and the possibility that the observed associations were influenced by residual or unmeasured confounding cannot be entirely excluded.

## Conclusion

Cognitive impairment was significantly associated with an increased risk of all-cause mortality among community-dwelling elderly Japanese men, independent of social engagement and other potential confounding factors. These findings suggest that cognitive impairment is an independent predictor of all-cause mortality in this population. Further prospective studies are warranted to clarify the causal relationships underlying this association.

## Declarations

### Availability of data and material

The data that support the findings of this study are not publicly available due to privacy reasons.
